# *Anisakis* extracellular vesicles elicit immunomodulatory and potentially tumorigenic outcomes on human intestinal organoids

**DOI:** 10.1186/s13071-024-06471-7

**Published:** 2024-09-17

**Authors:** Ilaria Bellini, Daniela Scribano, Cecilia Ambrosi, Claudia Chiovoloni, Silvia Rondón, Annamaria Pronio, Anna Teresa Palamara, Agostina Pietrantoni, Anna Kashkanova, Vahid Sandoghdar, Stefano D’Amelio, Serena Cavallero

**Affiliations:** 1 Department of Public Health and Infectious Diseases, Sapienza University of Rome, Rome, Italy; 2 Department of Human Sciences and Promotion of the Quality of Life, San Raffaele Open University, IRCCS, Rome, Italy; 3 Laboratory of Microbiology of Chronic-Neurodegenerative Diseases, San Raffaele Open University, IRCCS, Rome, Italy; 4 Digestive Endoscopy Unit, Department of General Surgery and Surgical Specialties “Paride Stefanini”, Sapienza University of Rome, Azienda Policlinico Umberto I, Rome, Italy; 5 Department of Infectious Diseases, Istituto Superiore di Sanità, Rome, Italy; 6 Core Facilities, Istituto Superiore di Sanità, Rome, Italy; 7 Max Planck Institute for the Science of Light, Erlangen, Germany; 8 Department of Public Health and Infectious Diseases, Sapienza University of Rome, Laboratory Affiliated to Pasteur Institute, Fondazione Cenci Bolognetti, Piazzale Aldo Moro 5, 00185 Rome, Italy

**Keywords:** Anisakiasis, Extracellular vesicles, Tumorigenic potential, Immunomodulation, Human intestinal organoids, Inflammation

## Abstract

**Background:**

*Anisakis* spp. are zoonotic nematodes causing mild to severe acute and chronic gastrointestinal infections. Chronic anisakiasis can lead to erosive mucosal ulcers, granulomas and inflammation, potential tumorigenic triggers. How *Anisakis* exerts its pathogenic potential through extracellular vesicles (EVs) and whether third-stage infective larvae may favor a tumorigenic microenvironment remain unclear.

**Methods:**

Here, we investigated the parasite's tumorigenic and immunomodulatory capabilities using comparative transcriptomics, qRT-PCR and protein analysis with multiplex ELISA on human intestinal organoids exposed to *Anisakis* EVs. Moreover, EVs were characterized in terms of shape, size and concentration using classic TEM, SEM and NTA analyses and advanced interferometric NTA.

**Results:**

*Anisakis* EVs showed classic shape features and a median average diameter of around 100 nm, according to NTA and iNTA. Moreover, a refractive index of 5–20% of non-water content suggested their effective biological cargo. After treatment of human intestinal organoids with *Anisakis* EVs, an overall parasitic strategy based on mitigation of the immune and inflammatory response was observed. *Anisakis* EVs impacted gene expression of main cytokines, cell cycle regulation and protein products. Seven key genes related to cell cycle regulation and apoptosis were differentially expressed in organoids exposed to EVs. In particular, the downregulation of EPHB2 and LEFTY1 and upregulation of NUPR1 genes known to be associated with colorectal cancer were observed, suggesting their involvement in tumorigenic microenvironment. A statistically significant reduction in specific mediators of inflammation and cell-cycle regulation from the polarized epithelium as IL-33R, CD40 and CEACAM1 from the apical chambers and IL-1B, GM-CSF, IL-15 and IL-23 from both chambers were observed.

**Conclusions:**

The results here obtained unravel intestinal epithelium response to *Anisakis* EVs, impacting host’s anthelminthic strategies and revealing for the first time to our knowledge the host-parasite interactions in the niche environment of an emerging accidental zoonosis. Use of an innovative EV characterization approach may also be useful for study of other helminth EVs, since the knowledge in this field is very limited.

**Graphical Abstract:**

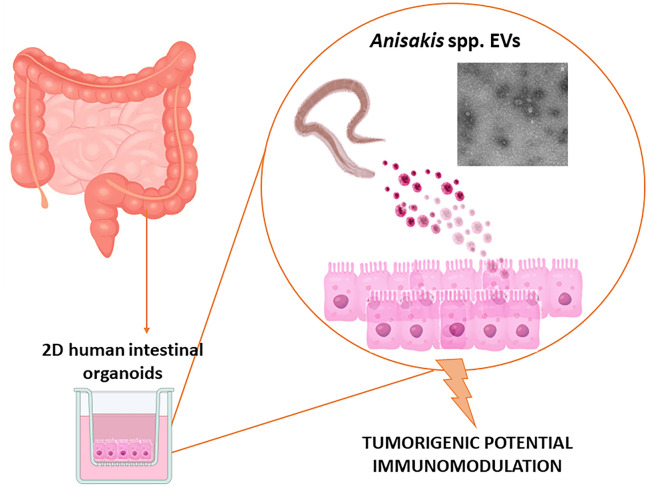

**Supplementary Information:**

The online version contains supplementary material available at 10.1186/s13071-024-06471-7.

## Background

Anisakids are cosmopolitan marine parasitic nematodes causing an emergent fish-borne zoonosis called anisakiasis or anisakidosis in humans that accidentally eat raw or undercooked seafood with the infective third-stage larvae (L3) [1]. To date, two *Anisakis* species have been frequently reported in human infections, *Anisakis simplex* sensu stricto and *Anisakis pegreffii *[2]. *Anisakis* L3 somatic and excretory/secretory products (ES) can determine a panel of gastrointestinal and/or hypersensitivity symptoms, with features of acute and chronic inflammation [3]. In physiological conditions, the inflammatory process promotes tissue and function restoration, but prolonged reactions due to the continuous triggering by antigens or persistent pathogens can lead to chronicity, often associated with an immunosuppressive state [4]. This, in turn, may favor a tumor microenvironment, characterized by suppressor-gene inactivation, oncogene activation and somatic mutations [5].

Helminths can survive for years into natural hosts by modulating the immune system and physiological state, and ES products can be related to pathogenesis, disease progression and even tumorigenesis [6]. The recent discovery of extracellular vesicles (EVs) as an additional mechanism to deliver messages between cells and organisms even across kingdoms of life, together with their detection in helminths, has changed the paradigm in the study of host-parasite interactions.

Nematodes such as *Heligmosomoides polygyrus* [7], *Brugia malayi* [8], *Ascaris suum* [9], *Nippostrongylus brasiliensis* [10] and *Trichuris muris* [11] produce EVs with immunosuppressive and immunoregulatory abilities. Helminth EVs may even be involved in tumorigenesis as demonstrated for carcinogenic flatworms [12, 13], but similar abilities in nematodes are not well understood. A correlation between nematode infections and cancer has been suggested for chronic infections caused by *Strongyloides stercoralis* [14], by *T. muris* in mice and in in vitro models [15] and by *Spirocerca lupi*, a parasitic nematode of canids causing malignant esophageal nodules [16]. Contrarily, *Trichinella spiralis* showed in vitro and in vivo antitumor activities [17]. In the framework of anisakiasis, reports showing gastric and colon tumors, colonic polypoid lesions and the co-occurrence of *Anisakis* L3 are increasing, with a described tendency of L3 to adhere to ulcerous, lesioned and cancerous mucosa [18, 19]. Previous exposure to *Anisakis* is a suggested risk factor for gastric or colon adenocarcinoma [20]. A predisposition to tumorigenic microenvironment in anisakiasis was observed in vitro [21]. As parasitic nematodes showed an unclear role in tumorigenesis, investigations on *Anisakis* pathogenetic mechanisms and carcinogenic potential are much needed.

The organoid model is the most advanced and powerful tool to study the pathogenesis of infectious agents. Organoids are multicellular bi- and tri-dimensional structures able to recapitulate the physiologic functions of the organ/tissue of origin, first established with murine intestinal epithelium and later also largely characterized in human tissue [22, 23]. They are gaining popularity for studying parasitic protozoan infections, such as toxoplasmosis [24]. Contrarily, organoids have been barely used in studies involving helminths but they could represent a suitable model for investigating several aspects spanning from nematode life cycle and development to interactions with hosts [25].

The aim of the present study was to explore the impact of *Anisakis* EVs on healthy human intestinal organoids (hereafter HIO). Comparative transcriptomics, along with gene expression and protein estimations, demonstrated significant differences in the expression of key genes related to cell cycle regulation and apoptosis in organoids exposed to *Anisakis* EVs, thereby suggesting their potential involvement in the development of a tumorigenic microenvironment. Moreover, a general mitigation of host immune response emerged. Our results show, for the first time to our knowledge, that human colonic organoids faithfully reproduce critical interactions between *Anisakis* L3 and the in vivo intestinal epithelial barrier. This validation underscores the potential of this novel technology for unraveling the intricacies of parasitic helminth infections with significant public health implications.

## Methods

### Parasite sampling, EV isolation and characterization

*Anisakis spp.* L3 were collected from the visceral cavity of 25 European hakes *Merluccius merluccius* and 60 anchovies *Engraulis encrasicolus* from area FAO 37 (Mediterranean Sea) between 2021 and 2022. Live L3s were incubated to collect EVs and then identified at species level, according to published methods [26, 27]. In brief, 470 L3 (6 pools with 20 larvae and 7 with 50 larvae) were incubated in RPMI (Gibco, containing biotin, vitamin B12, PABA, inositol, choline, Phenol Red) with Pen/Strep (1:100) for 24 h at 37 °C and 5% CO_2_. From these, a subsample of 120 L3 was subsequently identified to estimate species composition. The incubation media were stored at – 80 °C until used to isolate the EVs, with a precipitation-based approach (ExoQuick kit, System Biosciences, Palo Alto, CA, USA), according to MISEV2023 guidelines [28]. The samples obtained were eluted in 0.22-µm filtered PBS, mixed and immediately used for the treatment of HIO and for EV characterization of shape, size and concentration.

Classic nanoparticle tracking analysis (NTA), interferometric nanoparticle tracking analysis (iNTA), and scanning and transmission electron microscopy (SEM and TEM) were used.

NTA reads were performed with a Nanosight NS300 (Malvern Panalytical, Malvern, UK) according to our previous study [27]. iNTA coverglass preparation is according to Kashkanova et al. [29], and further experimental details are available as Additional file 1 (Additional Method 1). The measurement and analysis procedure is the same as previously described [30]. iNTA has an increased sensitivity to smaller particles missed by conventional NTA and measures the effective refractive index (RI), which is a function of particle composition. In fact, an EV can be modeled as a sphere surrounded by a thin shell (lipid bilayer). As the particle size increases, the influence of the shell decreases and the effective RI approaches the RI of the inner sphere. This can be used to estimate the amount of non-water content, which is extremely interesting as it reflects whether EVs are empty or not.

For SEM, EVs were left to adhere to polylysine-treated round glass coverslips (Ø10 mm). Samples were fixed with 2.5% glutaraldehyde in 0.1 M Na-cacodylate buffer and processed as previously described, with slight modification [31]. Briefly, samples were postfixed with 1% OsO4 in 0.1 M sodium cacodylate buffer and dehydrated through a graded series of ethanol solutions (from 30 to 100%). Then, absolute ethanol was gradually substituted by a 1:1 solution of hexamethyldisilazane (HMDS)/absolute ethanol and successively by pure HMDS until evaporation. Dried samples were mounted on stubs, coated with gold (10 nm), and analyzed in a GeminiSEM 450 (Carl Zeiss). For TEM negative staining, EVs were deposited on carbon-coated grids for electron microscopy. Phosphotungstic acid 2% and ammonium molybdate 4% (1:1 ratio) were added on grids for contrast. Samples were air-dried and observed at 100 Kw with a Philips EM208S (FEI-Thermo Fisher) and a Megaview II SIS (Olympus). A semiquantitative analysis was performed by measuring the diameters of at least 150 vesicles per sample using open-source Fiji software [32].

The protein concentration of the EV samples was evaluated using a Qubit4 according to the manufacturer’s instructions (Thermo Fisher Scientific, Waltham, MA, USA).

All relevant data of our experiments were submitted to the EV-TRACK knowledgebase (EV-TRACK ID: EV240048) [33].

### Human intestinal organoids

#### Three- and two-dimensional cultures

Three biological samples of HIO were used to test the effect of *Anisakis* L3 EVs. Two independent experiments were carried out, and each biological sample had technical duplicates. Samples were obtained after the acceptance of the ethical committees of the Policlinico Umberto I teaching hospital (protocol number 23882/2021). Colon organoid isolation, cultivation and treatment were performed at the Organoid laboratory of the Department of Public Health and Infectious Diseases of the Sapienza University of Rome. Briefly, colon biopsies were performed using endoscopic forceps, and crypts were isolated by washing the colonic tissue with cold DMEM/F12 (Corning, containing 1:1 Dulbecco's Modified Eagle Medium and Ham's F-12 media) and incubated with 10 mM EDTA for 30 min. Crypts were seeded in 50% Matrigel (Corning®, Kaiserslautern, Germany) in 24-well plates (Corning®). Growth medium (IntestiCult™ Organoid Growth Medium STEMCELL Technologies, Vancouver, Canada) was further supplemented with Pen/Strep (1:100) and gentamicin (1:1000). HIOs were incubated in a humidified chamber with 5% CO_2_ at 37 °C. The medium was refreshed every 2–3 days, and HIOs were passed every 7 days. After two passages, 3D organoids were mechanically dissociated and cultured in transwells generating 2D self-organizing structures that still recapitulate intestinal epithelia cell composition and spatial organization to allow the contact at the apical side with *Anisakis* EVs. Cells were counted and 1 × 10^5^ cells were seeded on Matrigel precoated Transwells® (Corning®; diameter: 12 mm; pore size: 0.4 μm). The basolateral chamber was filled with 0.6 ml growth medium, the apical chamber with 0.1 ml growth medium. After 14 days, the growing medium was replaced by the differentiation medium in both chambers (IntestiCult™ Organoid Differentiation Medium STEMCELL Technologies) for another 7 days until EV treatment. Two-dimensional intestinal organoids were treated every 24 h (two times with *Anisakis* EV-enriched fraction, controls were treated with only PBS), and after 48 h cells and media from both chambers were collected. Hereafter, controls are indicated as HOC and EV-treated organoids as HOT.

#### Two-dimensional human intestinal monolayer characterization with immunostaining

Aiming to verify and confirm the differentiation state of the intestinal model before the contact with *Anisakis* EVs, 2D organoids subsamples were washed with PBS and fixed in 4% paraformaldehyde (Santa Cruz Biotechnology) for 20 min at room temperature, then washed with PBS and stored at 4 °C until stained. Cell monolayers were permeabilized with 0.5% TRITON-X (Sigma) in PBS and blocked with 1% bovine serum albumin (BSA, Sigma) and 3% normal goat (Gibco) in PBS for 2 h at room temperature. Cells were washed once with PBS and incubated overnight at 4 °C with mouse anti-human ZO-1 (1:500, 33-9100, Invitrogen, Thermofisher) and mouse anti-human Villin (1:250, sc-58897, Santa Cruz Biotechnology) monoclonal antibodies and rabbit anti-human MUC2 (1:250, PA5-21329, Invitrogen, ThermoFisher) polyclonal antibody. All primary antibodies were diluted in PBS containing 1% BSA. After washing three times with PBS with 0.1% TRITON-X, cells were incubated for 1 h at room temperature with 1:250 diluted rhodamine (TRITC)- and fluorescein (FITC)-conjugated anti-mouse and anti-rabbit secondary antibodies (Jackson ImmunoResearch), respectively. Cells were washed three times with 0.1% TRITON-X in PBS, stained with DAPI (2 µg/ml, Invitrogen) for 10 min at room temperature and then washed three times. Membranes were placed onto a glass slide and then stored at 4 °C until processing. Images were recorded with a Leica DM5000B microscope equipped with DFX340/DFX300 camera and processed using the Leica Application Suite 2.7.0.R1 software (Leica).

### Comparative transcriptomics and qRT-PCR

After 48 h of treatment with *Anisakis* EVs, total RNA was isolated using TRIsure™ reagent (Bioline, London, UK) from monolayers and tested for the amount and quality of RNA obtained, according to a previous work [34]. The material was used for library preparation for the RNA-seq and cDNA synthesis to perform qRT-PCR. Regarding RNA-seq, 2 μg was used for RNA-seq, and the Universal Plus mRNA-Seq kit (Tecan Genomics, Redwood City, CA) was used for library preparation following the manufacturer’s instructions. RNA samples and libraries were also tested by Agilent 2100 Bioanalyzer RNA assay (Agilent Technologies, Santa Clara, CA) and by Caliper LabChip GX (PerkinElmer, Waltham, MA). Libraries were sequenced on paired-end 150-bp mode on NovaSeq 6000 (Illumina, San Diego, CA).

Raw reads were used for base calling, demultiplexing and adapter masking with Illumina BCL Convert v3.9.31 and then trimmed by ERNE2 software [35]. Reads were analyzed for statistics on “strandness” of reads, gene-body coverage, reads distribution and insert size using the RSeqQC5 package [36]. The high-quality pair-end reads were aligned to the reference genome (human genome version hg38-iGenomes) with STAR3 [37]. Assembling and quantification of full-length transcripts representing multiple spliced variants for each gene locus were obtained using Stringtie4 [38]. Pair-wise differential expression analysis transcripts (HOC vs. HOT) were performed using the htseq-count6 package [39] by counting the overlap of reads with genes and with DESeq2 package [39, 40] to compare expression levels of genes and transcripts by fitting a generalized linear model (GLM) for each gene. Normalization was performed using the median-of-ratios method [41]; statistical significance was determined using a Wald test (FC > 1; FDR < 0.05). Correlation of all samples/groups with VST-normalized data and FPKM-normalized data was obtained using PCA. The list of differentially expressed transcripts (also commonly indicated as differentially expressed genes, DEGs) was further analyzed, including three categories to explore the effect of *Anisakis* EVs on HIO: transcripts with significant FDR; up- and downregulated transcripts in HOT (without significant FDR but with log2FC > 2). These were characterized for Gene Ontology using Gene Ontology web resource and Panther v17.0 (Fisher exact test and FDR with *P* < 0.05) in terms of molecular pathways using Genecards and Uniprot as well as String resource to gain a functional assessment of the potential interactions of transcripts.

Significant DEGs and transcripts in HOT with log2FC > 2 were also checked as putative gene targets of the 13 EV-enriched *Anisakis pegreffii* miRNAs [27], using miRDB custom search (with prediction score > 80 considered real, according to Liu and Wang [42]; Chen and Wang [43] and Targetscan 8.0 [44]). A list of genes of interest was evaluated for the relative quantification by real-time PCR to validate the bioinformatics data and to explore the expression trend of immunomodulatory factors in HOT. A total of 1 μg RNA for reverse transcription of each sample for qRT-PCR, using SuperScript II RT and OligodT (Invitrogen, Waltham, MA, USA) according to the manufacturer’s protocol. The following genes were studied: *Il1β, Il8, Il33, NUPR1, EPHB2, LEFTY, TACC1. GAPDH* was used as endogenous control (list of primers is available in Additional file 1, Table S3). Amplification protocol and data analysis including relative quantification of transcripts using delta delta Ct are according to Bellini et al. [34].

### Cytokines and other protein measurements

Multiplex assay for cytokines and other factors of interest was performed on the following analytes: GM-CSF, IL-1B/IL-1F2, IL-2, IL-6, IL-13, IL-15, IL-17/IL-17A, IL-22, IL-23, IL-33, CD40/TNFRSF5, CEACAM-1, CEACAM-5, CXCL5/ENA-78, IL-8/CXCL8, IL-10, ST2/IL-33R. Bio-Plex Magpix Multiplex Reader and the associated software were used (BioRad). The assay is capable of simultaneously quantifying several targets providing more information from a lower sample volume in less time than traditional immunoassay methods, using differentially detectable bead sets and fluorescence. Here, a total of 17 analytes were selected, of which 7 processed with a standard protocol and 10 for a high-sensitivity protocol, intended for low-amount targets (Biotechne R&D Systems Luminex Discovery assay Human Premixed Multianalyte Kit and Human HS Cytokine Kit). The concentration of each target was measured regarding the calibration curve (individual for each target and realized using standards provided by the company). The experimental steps were according to the manufacturer’s instruction, using 50 ul of supernatants from apical and basal chambers, evaluated separately, considering the epithelium polarity. Data are expressed as pg/ml according to internal standard controls and reported as mean ± SEM (standard error mean). Significance was evaluated using a Student’s t-test pairing for controls vs. treated, with *P* < 0.05.

## Results

### *Anisakis* species composition and characterization of EVs

Parasitic EVs to treat human intestinal organoids were obtained by incubating *Anisakis* L3 in pools of 20 and 50 nematodes at 37 °C. A subsample of *Anisakis* L3 (*n* = 115) was identified at the species level to depict the relative frequencies of different species potentially present in the sample, and *A. pegreffii* was the prevalent species, as expected being the most widespread species in the Mediterranean Basin, with the highest frequency among specimens (91.3%). Other species occurring in the same area were also identified: *Anisakis simplex* s.s. (0.9%), the hybrid genotype of the two sibling species (5.2%), and *Anisakis physeteris* (2.6%).

**Fig. 1:**
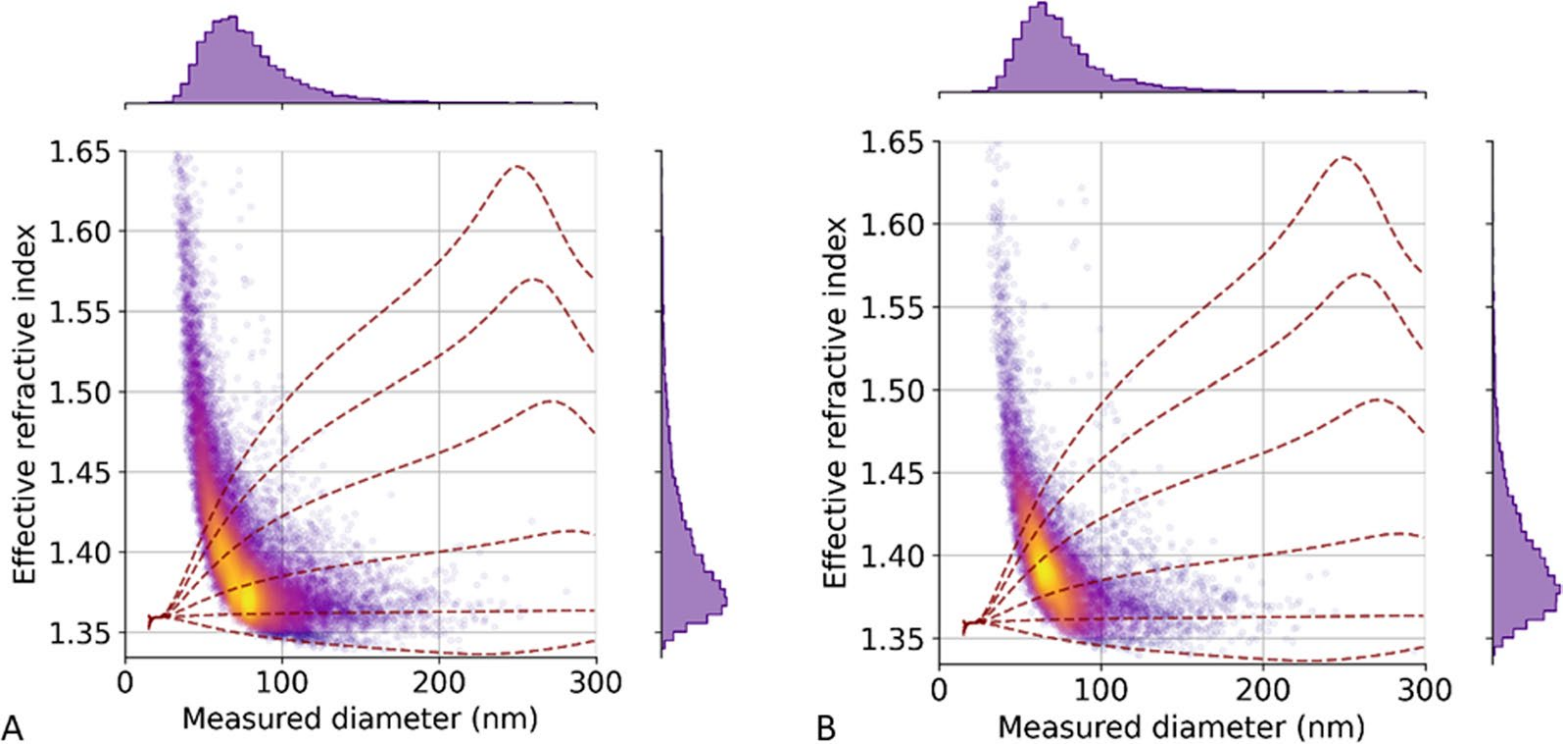
iNTA scatter plot of the *Anisakis* EVs accounting for effective RI and diameter (**A** pool of 50 L3, **B** pool of 20 L3). Color bar denotes point density. The red lines correspond to a shell thickness of 5 nm and shell refractive index of 1.44. The inner protein (non-water) content varies from the bottom to the top line as 0%, 10%, 25%, 50%, 75% and 100%

The protein concentration observed in *Anisakis* EVs was around 80 ng/ul, and 3.2 µg protein was used during model treatment. EV size and shape were obtained by NTA, iNTA, SEM and TEM. NTA reported an average median diameter of 145.5 nm and 1.64 × 10^10^ particles/ml. In comparison, iNTA revealed a smaller median size (69, 6 nm) and a higher number of particles/ml (3 × 10^11^), likely due to increased sensitivity to smaller particles missed by conventional NTA. NTA and iNTA data are available in Supplementary information (Additional file 1: Table S1; Figure S1 and S2). In addition, iNTA measures the effective refractive index (RI), which is a function of particle composition. In Fig. 1, the RI is between 1.35 and 1.4 for particles > 100 nm, corresponding to 5–20% non-water content, consistent with estimates previously obtained for EVs of *Leishmania* parasites[30]. Since the distribution of the effective RI of the particles is quite broad, there is likely to be a large variation in both the amount of non-water content and the shell parameters. To better understand this, lines of a constant amount of non-water content (0%, 10%, 25%, 50%, 75%, 100%) were plotted, assuming the same parameters for the particle shell as extracted for *Leishmania* EVs (shell RI 1.44, shell thickness 5 nm). Lines of a constant amount of non-water content assuming a shell consisting of loose protein corona with 10 nm thickness and 1.36 refractive index are available in Figure S2. Note that the large distribution of effective RI for smaller particles is in part caused by the uncertainty in contrast determination[29].

**Fig. 2:**
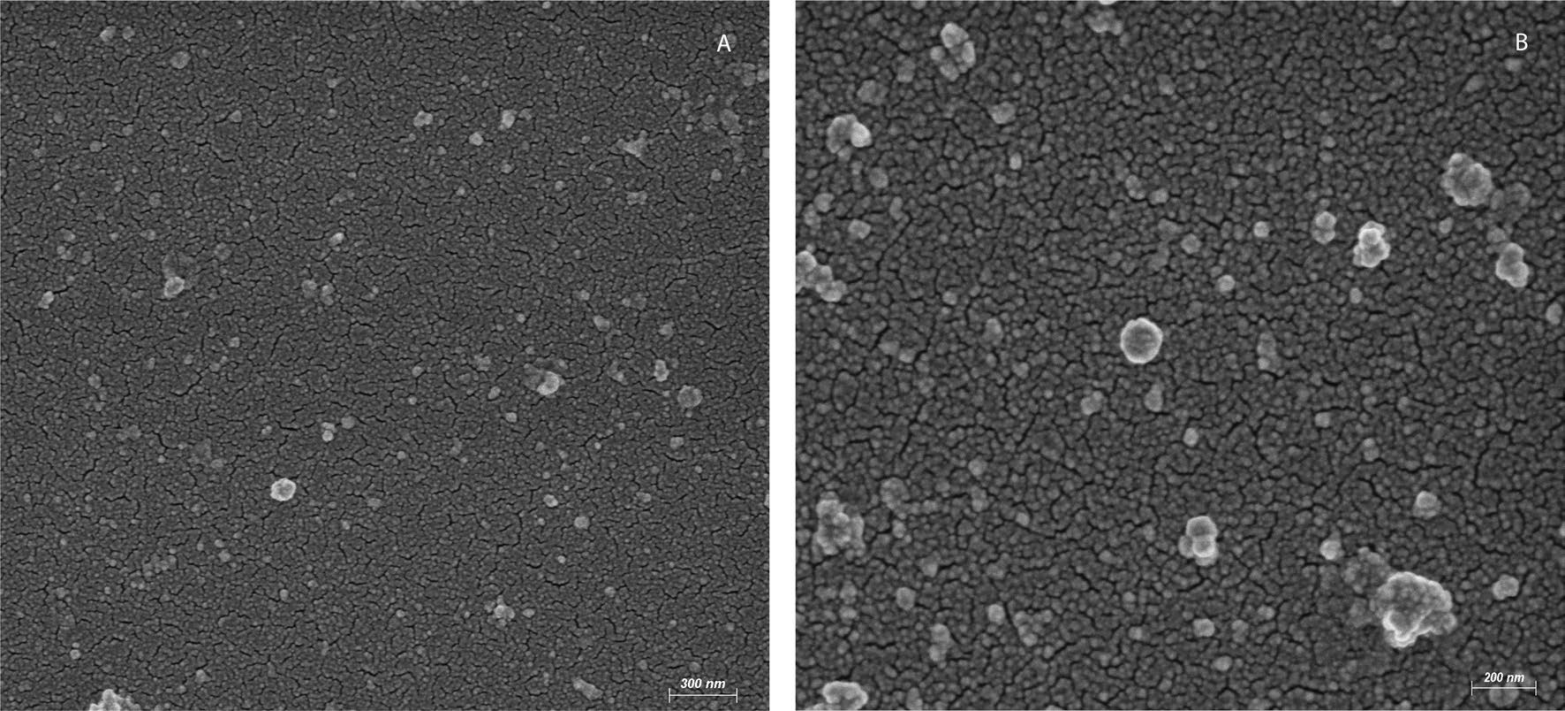
Scanning electron microscopy of *Anisakis*-derived extracellular vesicles (EVs). EV-like structures (range: 80–200 nm) released in culture media by *Anisakis* L3 (pool samples of 50 L3 in **A** and of 20 L3 in **B** after 24 h incubation and isolation visualized by SEM. Scale bar is indicated (**A** 200 nm; **B** 300 nm)

**Fig. 3:**
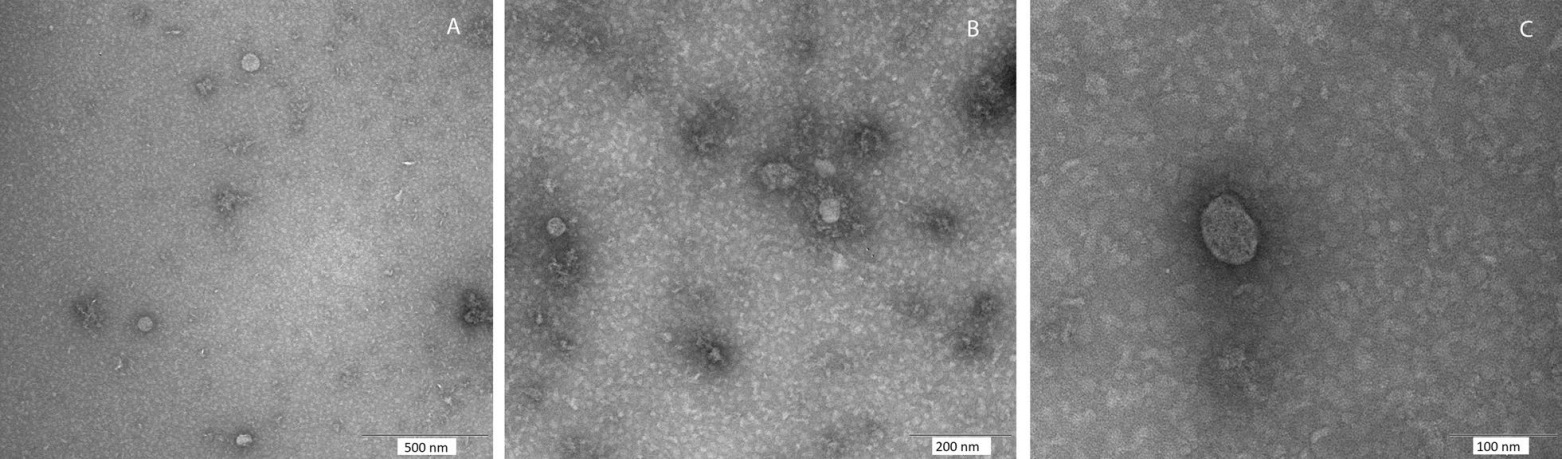
Transmission electron microscopy of *Anisakis*-derived extracellular vesicles (EVs). EV-like structures released in culture media by *Anisakis* L3 (a mix of the two pool samples of 50 and 20 L3). Blackish area surrounding EVs indicative of protein corona is visible. Scale bars are indicated (A: 500 nm; B: 200 nm; C: 100 nm)

SEM observation showed two preparations that consisted prevalently of vesicles (Fig. 2), and TEM characterization confirmed the lipidic membrane-enclosed nature of particles (Fig. 3), with putative external molecules (i.e. glycans and lipids) and corona proteins visible as a possible feature of EVs. EV particles showed a size range of 80–200 nm in diameter, with a median value of 71 nm and IQR of 42–120 nm. The three distributions of diameters values collected from TEM, NTA and iNTA are available in Additional file 1: Figure S3.

Given that size and concentration of EVs obtained from the two pools were comparable, the enriched fractions obtained were mixed before the administration to the in vitro model. The average number of particles for NTA and iNTA allowed estimating a range of *Anisakis* EVs administrated per cell, corresponding to 3 × 10^3^–6 × 10^3^ particles.

### *Anisakis* EVs alter intestinal cell gene expression

**Fig. 4:**
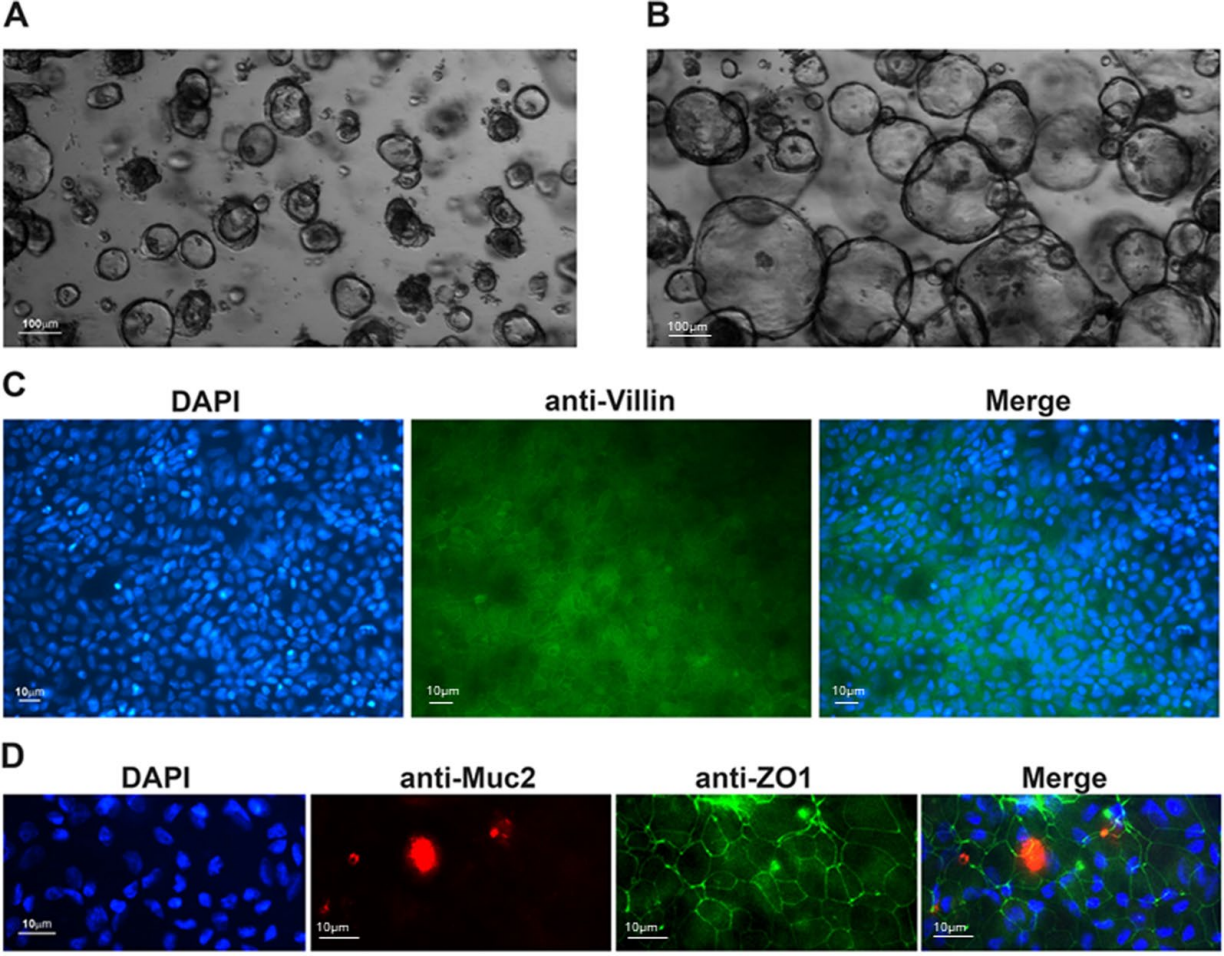
Human intestinal monolayer cultures obtained from intestinal organoids with differentiated cell types and junctional complexes. **A**, **B** Intestinal organoids isolated from donor colonic tissues and cultured in Matrigel; representative light microscopy images acquired after 3 days **A** and 10 days **B** of growth. Scale bar: 100 µm. **C**, **D** At 14 days of culture, intestinal epithelial cell monolayers were stained with specific antibodies to visualize enterocytes (Villin, green), goblet cells (Muc2, red) and tight junctions (ZO1, green). Scale bar: 10 µm. In all immunofluorescence images DAPI was used to stain nuclei

To evaluate the impact of *Anisakis* EVs on functional human intestinal tissue, intestinal organoids were obtained starting from colonic biopsies from three healthy donors, corresponding to three biological samples. Three-dimensional cultured organoids were used to amplify intestinal cells (Fig. 4A, B), while 2D cultures were used to generate the differentiated intestinal tissue (Fig. 4C, D) and perform treatment with *Anisakis* EVs every 24 h with two administrations.

After 48 h of treatment, 2D organoids and supernatants (apical and basolateral chambers separated) were collected for downstream analyses. Total RNAs from 2D human intestinal organoids were used for transcriptome analyses. An average of 87 million and 60 million 150-bp reads were generated for HOC and HOT, respectively (Additional file 1: Table S2). Raw reads were submitted to the Sequence Read Archive (SRA) database of NCBI (http://www.ncbi.nlm.nih.gov/sra) under Bioproject PRJNA942614. Sample correlation supported the expected variation among the three different biological samples (Additional file 1: Figure S4). Normalizations and quantitative estimations were performed on around 16 K unique transcripts.

The top100 most abundant transcripts were involved in “structural molecule activity” (GO:0005198) as ribosomal proteins, in “binding” (GO:0005488) and “catalytic activity” (GO:0003824) as chaperones (Hsps) and major histocompatibility complex proteins (MHCs), crucial for the positive regulation of adaptive immune response (HLA-A, B, C and B2M). Others are transcriptional fingerprints of a functional and differentiated colonic tissue or involved in the regulation of apoptotic signaling pathways (LGALS3, RPS3 and TMBIM6). Among the most represented protein classes in these highly abundant transcripts, translational (PC00263), ribosomal (PC00202) and cytoskeletal proteins (PC00085) were the most prevalent. Details on the top100 most abundant transcripts are available in Additional file 2.

The differential expression analyses provided some clues about the effect of *Anisakis* EVs on human intestinal organoids. We focused on transcripts with (i) significant FDR (*n* = 7) and (ii) non-significant FDR but with log2FC > 2 at pairwise comparison (*n* = 95 for upregulated and *n* = 210 for downregulated). The complete lists of up- and downregulated transcripts and gene ontology results are available in Additional file 3.

Interestingly, the 95 upregulated transcripts with log2FC > 2 in HOT were related to GPCR genes, involved in signal transduction and host-pathogen interactions; to RNA component of mitochondrial RNA replication; to RAET1 family, involved in antigen presentation (MHC I); to members of the lysyl oxidase gene family, essential to the biogenesis of connective tissue and implicated in oncogenesis and regulation of cell fate (WNT).

Regarding the 210 downregulated with log2FC > 2 in HOT, a role in cell division or apoptosis regulation was observed (i.e. EXO1, CDC25C, SKA and DDIAs). Considering the regulatory role in host genes of parasitic-derived miRNAs transported into *Anisakis* EVs, *Anisakis*-derived miRNAs selectively packaged into previously identified EVs [27] were used as input to investigate potential gene targets in HIO:10 putative genes (Table 1), and two significant DEGs downregulated in HOT were obtained. In particular, ape-lin-4-5p was associated with the putative gene target EPHB2 (score 68) and ape-miR-72-5p with the putative gene target TACC1 (score 81).

**Table 1:** List of putative gene target of *Anisakis* miRNAs enriched in EVs, selected among the human intestinal organoid transcripts. Information about Anisakis miRNAs, the human homologous miRNA, the putative gene target, the type of transcripts according to categories of interest (top100 most abundant, transcript with an increasing or decreasing trend or significant DEGs in human intestinal organoids treated with Anisakis EV compared to controls; *downregulated DEGs) and the putative role are reported

*Anisakis* miRNA	Human homologous	Putative gene target	Class of DEGs	Putative role
Ape-miR-7-5p	hsa-miR-7-5p	TFF3	Top100	Function poorly understood, they may protect the mucosa from insults, stabilize the mucus layer and affect healing of the epithelium
		LOXL2	Trend up	Member of the lysyl-oxidase gene family. The prototypic member of the family is essential to the biogenesis of connective tissue
Ape-novel-miR-57	hsa-miR-584-5p	EEF2	Top100	Essential factor for protein synthesis; related to peptide chain elongation and innate immune system pathways
Ape-miR-72-5p	hsa-miR-31-5p	HLA-A	Top100	RNA binding and peptide antigen binding
		CALR	Top100	Calreticulin involved in cell adhesion
		MYH9	Top100	Conventional non-muscle myosin, involved in cytokinesis, cell motility and maintenance of cell shape
		TACC1*	DEG down	Involved in transcription regulation induced by nuclear receptors. Breast cancer candidate gene
Ape-novel-miR-69	No matches	PGK1	Top100	Protein secreted by tumor cells, it participates in angiogenesis by functioning to reduce disulfide bonds in the serine protease, plasmin, which consequently leads to the release of the tumor blood vessel inhibitor angiostatin
Ape-novel-miR-184	hsa-miR-4448	MYH9	Top100	conventional non-muscle myosin, involved in cytokinesis, cell motility and maintenance of cell shape
		PABPC1	Top100	Regulates processes of mRNA metabolism such as pre-mRNA splicing and mRNA stability. Its function in translational initiation regulation
		RPL26	Top100	Component of the 60S subunit
Ape-novel-miR-65	No matches	HSP90AA1	Top100	Inducible molecular chaperone that promotes the maturation, structural maintenance and proper regulation of specific target proteins involved in cell cycle control and signal transduction; diseases associated with HSP90AA1 include hepatocellular carcinoma
		RPL27A	Top100	Part of ribosomal 60S Variable expression of this gene in colorectal cancers compared to adjacent normal tissues has been observed, although no correlation between the level of expression and the severity of the disease has been found
		GPR17	Trend up	G protein-coupled receptor activity and chemokine receptor activity
Ape-novel-miR-27	No matches	SCD		Fatty acid biosynthesis, primarily the synthesis of oleic acid of hemidesmosomes, multiprotein complexes at the dermal-epidermal basement membrane zone that mediate adhesion of keratinocytes to the underlying membrane
		COL17A1		Alpha chain of type XVII collagen, structural component of hemidesmosomes
Ape-novel-miR-19	hsa-miR-4502	CEP55		Plays a role in mitotic exit and cytokinesis
Ape-lin-4-5p	hsa-miR-7976	EPHB2*		Member of the Eph receptor family of receptor tyrosine kinase transmembrane glycoproteins. They bind ligands called ephrins and are involved in diverse cellular processes including motility, division, and differentiation. May function as a tumor suppressor
Ape-novel-miR-131	No matches	GPR17	Trend up	G protein-coupled receptor activity and chemokine receptor activity

The seven statistically relevant DEGs (Table 2) included two upregulated (NUPR1 and H2BC5) and five downregulated transcripts (LEFTY1, TACC1, MYBL2, MKI67 and EPHB2). *LEFTY1* is mainly involved in transmembrane receptor protein tyrosine-kinase signaling and cytokine activities, while *EPHB2* participates in TGF-β-PDGF signaling pathways. Cell cycle regulation and apoptosis were the main biological processes detected in this shortlist.

**Table 2:** List of seven significant DEGs, with indication about their regulation and biological features, according to the literature available in Genecards and Uniprot repositories

Gene ID	DEGs	Putative role—Genecards	Putative role—Uniprot
LEFTY1	Down	Encodes a secreted ligand of the TGF-beta protein superfamily; the mature protein plays a role in left-right asymmetry determination of organ systems during development	Required for left-right axis determination as a regulator of LEFTY2 and NODAL
MKI67	Down	Nuclear protein associated with and maybe necessary for cellular proliferation	Required to maintain individual mitotic chromosomes dispersed in the cytoplasm following nuclear envelope disassembly
EPHB2	Down	Member of the Eph receptor family of receptor tyrosine kinase transmembrane glycoproteins. They bind ligand ephrins involved in cellular processes including motility, division and differentiation	May function as a tumor suppressor: diseases associated with EPHB2 include bleeding disorder, platelet type 22 and prostate cancer/brain cancer susceptibility
TACC1	Down	Transforming acidic coiled-coil-containing protein or gastric cancer antigen Ga5. Breast cancer candidate gene	Involved in transcription regulation induced by nuclear receptors, including in T3 thyroid hormone and all-trans retinoic acid pathways
MYBL2	Down	Nuclear protein involved in cell cycle progression, it activates the cell division cycle 2, cyclin D1 and insulin-like growth factor-binding protein 5 genes	Transcription factor involved in the regulation of cell survival, proliferation and differentiation
NUPR1	Up	Acts upstream of or within negative regulation of cell cycle. Diseases associated with NUPR1 include pancreatic cancer	Transcription regulator that converts stress signals into a program of gene expression, which empowers cells with resistance to stress. Participates in regulation of cell cycle, apoptosis, autophagy and DNA repair responses
H2BC5	Up	This gene is intronless and encodes a replication-dependent histone that is a member of the histone H2B family	Plays a central role in transcription regulation, DNA repair, DNA replication and chromosomal stability

**Fig. 5:**
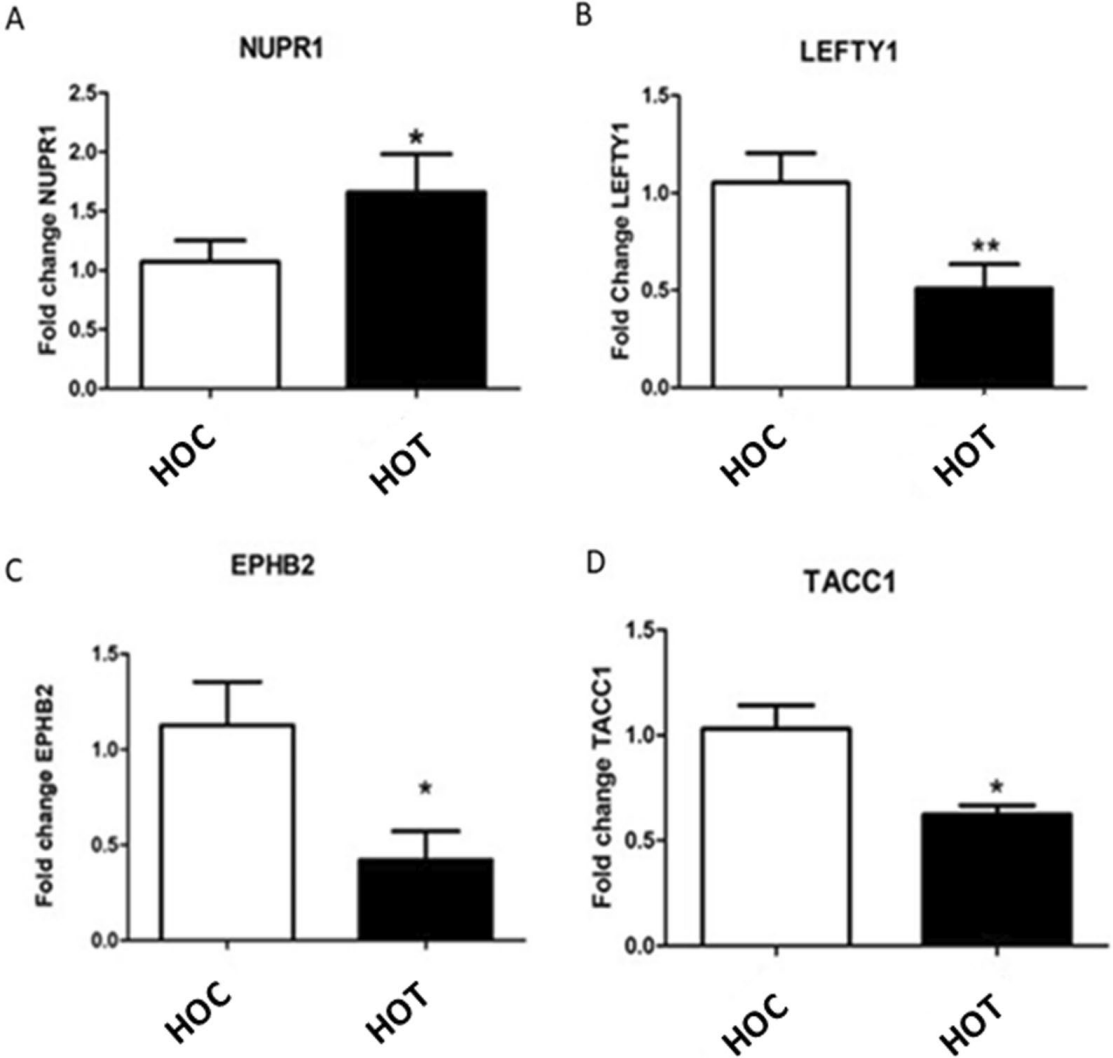
*Anisakis* extracellular vesicle (EV) modulation of genes in 2D cultures of human intestinal organoids after 48 h of exposure. **A** NUPR1 gene expression in HIO (*P* = 0.03). **B** LEFTY1 gene expression in HIO (*P* = 0.002); **C** EPHB2 gene expression in HIO (*P* = 0.02) and **D** TACC1 gene expression in HIO (*P* = 0.01). Data are expressed as a fold change compared to the control samples and as means ± SEM (standard error mean). Significance was evaluated using a Student’s t-test pairing for the HOC vs. the HOT

Relative quantifications of gene expression to confirm bioinformatics data using four representative DEGs in terms of expression trend and statistical significance in pairwise comparisons confirmed *NUPR1* was significantly increased (*P* = 0.03), while *LEFTY1, EPHB2* and *TACC1* were significantly decreased (*P* = 0.002, *P* = 0.02 and *P* = 0.01, respectively) in human intestinal organoids exposed to *Anisakis* EVs (Fig. 5).

### *Anisakis* EVs alter the expression of key cytokines involved in anti-helminthic strategy

**Fig. 6:**
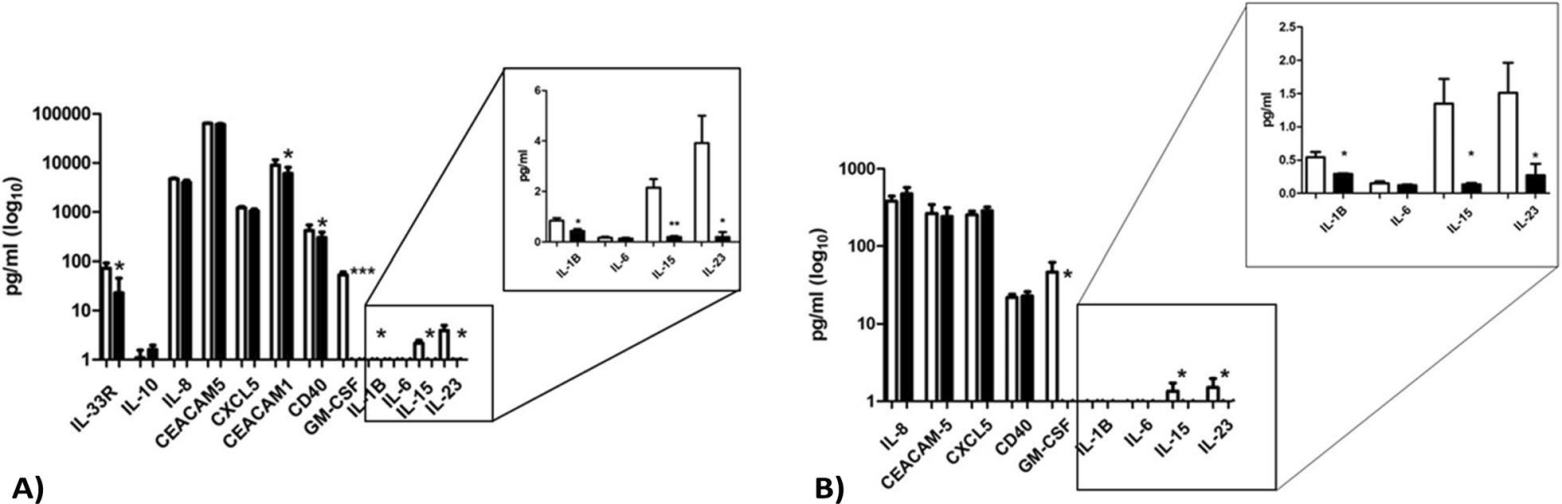
Luminex assay on selected cytokines and factors of interest. Material analyzed from supernatants of incubation collected from **A** the apical and **B** basal chambers of the human intestinal organoid controls (HOC white bars) and treated with *Anisakis*-derived EVs (HOT black bars), *P < 0.05

Given the influence of parasitic EVs on cellular responses to nematode infections, we selected specific mediators of inflammation and cell cycle regulation for protein estimation (Fig. 6). A significantly reduced concentration of IL-33R, CD40, CEACAM-1, IL-1B, GM-CSF, IL-15 and IL-23 was observed in the supernatant collected from the apical epithelial side of HOT (Fig. 6A). The same trend was observed for IL-1b, GM-CSF, IL-15 and IL-23 analyzed from the basal side (Fig. 6B). A lower amount compared to the apical chamber was observed for IL-8, CEACAM-5, CXCL-5 and CD40, while IL-6 and IL-33R, IL-10 and CEACAM-1 were not detected. The signals for IL-33, IL-17A, IL-13 and IL-22 were not detectable in either samples or chambers and therefore excluded from the results. Moreover, the expression of genes encoding for few selected inflammatory cytokines and immunomodulatory factors, such as IL-1b, IL8 and IL33, exhibited a not statistically decreasing trend in treated organoids (Additional file 1, Figure S5).

## Discussion

Helminths can live for a long time within their vertebrate hosts, thanks to immune cell signaling pathway manipulation, by directing a tolerant or hyporesponsive state through downregulation or inactivation of the innate and adaptive immune response, naturally directed to the helminth expulsion [45]. This scenario is not expected in anisakiasis, as interactions taking place between *Anisakis* and humans are not the result of natural co-evolutionary or co-adaptation processes. However, the close phylogenetic relation with the human ascaridoid *Ascaris* spp. and the common site of infection in the definitive hosts (i.e. the gastrointestinal tract) may suggest a common evolutionary strategy selected in the frame of interactions with definitive hosts, marine and terrestrial mammals. Since helminth EVs elicit diverse responses in their hosts, *Anisakis* EVs may also be involved in pathogenic conditions. Recent efforts in this field have described the ability of *Anisakis* L3 to produce vesicles, primarily originating from the excretory pore and anterior end [46, 47]. Here, four different approaches were used to characterize the shape, size and concentration of *Anisakis* EVs, confirming a predominance of typical microvesicles < 100 nm and suggesting the presence of surrounding external molecules and protein corona. Moreover, EV median size obtained from TEM analyses agreed with values from iNTA, according to their distribution. iNTA has been previously used only once in parasitology to investigate EVs released by *Leishmania* [30], and it was used here for the first time to our knowldge to analyze parasitic helminth-derived EVs. By using independent measurements of hydrodynamic size and scattering cross section, iNTA allows extraction of RI for individual particles and is able to resolve subpopulations of particles with different physical properties, as demonstrated for human biological samples of EVs with lipoproteins [29]. Similar to *Leishmania* EVs, we obtained an inner non-water content of 5–20%. However, as opposed to *Leishmania* EVs, we detected smaller particles and noticed a larger distribution of effective RIs. Due to the heterogeneity of particle populations, which is also evident across different isolation methods in helminths [48], an accurate estimation of the quantity and kind of administered EVs in the present model and generally in studies using parasite EVs is challenging. Therefore, we strongly advocate for the systematic collection of extensive data through several techniques, coupled with the use of state-of-the-art in vitro models. New optical approaches such as iNTA are also needed in parasitological studies to discriminate EVs from contaminants and to detect EV populations that differ in size. This, along with the discovery of specific markers, may support a future precise identification of packaged content according to different sizes of EVs and potentially trace specific biological effects. Such knowledge could in turn be applied in therapy or control of parasitic diseases.

To date, *T. muris*, *A. suum* and *N. brasiliensis* EVs have been microinjected into 3D murine and canine organoids, attempting to mimic parasite interaction with the apical side of host epithelium [10, 11, 49, 50]. Two-dimensional intestinal organoid cultures were also shown to be very useful to model the intestinal epithelium as they allow easy access to the apical side [25]. Here, we provide the first study analyzing the effect of zoonotic nematode-derived EVs on human intestinal organoids developed from colon tissues collected from healthy donors. In a previous work carried out on Caco-2 cells, a general immunosuppressive activity related to alive *Anisakis* L3 and EVs and a strong proinflammatory action of the dead parasites was shown [34]. Similarly, a downregulation of the type 2 innate immune response was observed in mice infected with intestinal roundworm *H. polygyrus*. In particular, secreted EVs promoted parasite survival by suppressing the expression of genes involved in inflammation and immunity, such as the IL33R and DUSP1, a key regulator of mitogen-activated protein kinase (MAPK) signaling [51]. Furthermore, *Heligmosomoides bakeri* induces hyperplasia in goblet and tuft cells and an increase in mucus and alarmin production to promote parasite expulsion by involving IL-4, IL-13 and IL-25. Moreover, increased IL-33 alarmin in turn drives the type 2 immune response [52]. Affecting type 2 immune response could represent a common strategy among nematodes to survive within their hosts, as also shown in the present study, in which a significant reduction in IL-33R from the apical chambers and decreasing trend in IL33 gene expression in 2D organoids were observed in the presence of *Anisakis* EVs.

Besides their main immunomodulatory activity, cytokines also play an important role in pro- or anti-tumor activity [53]. GM-CSF is expressed at a low level by epithelial cells of healthy human colon in vivo, by colon cancer cell lines and human colon cancer biopsies, and in mucosal lesions of inflammatory bowel disease patients [54]. Identified as a key mediator of chronic inflammation, GM-CSF shows both inflammatory and immunosuppressive activity, depending on the dose and overall cytokine panel [55]. In spirocercosis, it showed downregulation in the neoplastic group compared to the non-neoplastic group [16]. Here, a significant reduction in GM-CSF in both chambers was observed after *Anisakis* EV treatment, according to a previous study using whole-live *Anisakis* L3 [56]. Hence, by acting on one of the most important activators of macrophages, *Anisakis* EVs could contribute to downregulating the immune response.

While the dysregulation of IL-6 plays a crucial role in chronic helminthiasis, also potentially related to cancer [12], IL-6's role in anisakiasis is less clear. A strong IL-6 downregulation was previously reported after treatment with *Anisakis* EVs in Caco2 cells [34] not detected here using the healthy colonic model, while a significant decrease of IL-15, IL-23 and IL-1B was observed in both chambers (*p* = 0.05). IL-15 is involved in the innate response by activating natural killer cells, and it can also be stimulated by GM-CSF [57]. The inflammatory cytokine IL-23 is involved in Treg-type response by activating Th-17 cells, which in turn activate GM-CSF to mediate protection against extracellular pathogens and participate in barrier immunity. Interestingly, an impairment in IL-23 and IL-33R has been associated with inflammatory bowel disease (IBD) [58]. GM-CSF is also involved in IL-1B secretion, an important mediator of the inflammatory response, associated with cell proliferation, differentiation and apoptosis. Moreover, its dysregulation correlates with gastric cancer [59].

A significant decrease in the level of CD40 was also observed at the apical chamber (*P* = 0.05). It is an essential receptor member of the TNF family for T cell-dependent immunoglobulin class switching and memory B cell development. It is also expressed in non-immune cells and tumors [60], and its downregulation may suggest a further impairment of a successful adaptive immune response. A potential cancer-related immunomodulatory action of *Anisakis* EVs also emerged with reduction in CEACAM-1 protein (*P* = 0.01), which mediates angiogenesis, inflammation and innate and adaptive immune responses [61]. CEACAM-1 downregulation is reported in > 85% of early colorectal adenomas and carcinomas; vice versa, its overexpression is detected in advanced stages of malignancies [62]. The induction of immunosuppression promotes tolerance and a longer host-parasite interaction, potentially leading to a tumorigenic microenvironment, often associated with gene dysregulation, in which also cytokines play a key role. Downregulation of inflammatory cytokines can lead to an immune-suppressive environment that supports tumor growth by reducing the immune system's ability to fight off neoplastic changes. Therefore, chronic inflammation and prolonged host-parasite interaction, combined with the silencing of key cytokines, can create a permissive environment for tumor development by combining promotion of cellular instability and weakening the immune system's ability to respond to these changes. Upregulation of *NUPR1* and *H2BC5* genes has been associated with various cancer types. *NUPR1* was characterized in colorectal cancers, where it exhibits high expression in the primary tumor stage [63]. *H2BC5* is a member of the H2B histone family usually upregulated in cholangiocarcinoma and esophageal carcinoma, among many others. The downregulation of *EPHB2,* involved in cell cycle processes, and *LEFTY1*, involved in intestinal mucosal immunity and TGF-β signaling, is a common feature also observed in colorectal cancers and Crohn’s disease, respectively [64, 65]. However, *MYBL2* and *MKI67* are usually upregulated in different tumors, but recent studies showed that the downregulation of their mRNA is mediated by the P53-P21 pathway [66, 67]. A further clue derives from the positive match of downregulated human transcripts and *Anisakis* miRNAs enriched in EVs, highlighting the potential biological role of two abundant miRNAs. *Anisakis* ape-miR-72-5p is orthologous of human hsa-miR-31-5p, which showed both oncogenic and/or tumor suppressor activities. Its upregulation correlates with sporadic early onset of colorectal cancer [68], and its expression level is a potential prognostic factor for colon adenocarcinoma [69]. *Anisakis* ape-lin4-5p was predicted to target other genes involved in cell cycle regulation, apoptosis and inflammation, as FREM1 is also known as TILR for Toll-like/interleukin-1 receptor regulator-coreceptor of interleukin 1 [27].

## Conclusion

The present study explores the interaction between the natural cellular niche in which anisakiasis takes place thanks to using an ex vivo cutting-edge model based on human intestinal colon-derived organoids and parasitic EVs. Evidence suggests that *Anisakis* EVs may be involved in the potential induction of a tumorigenic microenvironment caused by the dysregulation of key genes and mitigation of immune response. Considering the rising number of case reports in the literature describing the co-occurrence of tumorigenic lesions and anisakiasis, further investigations are needed to characterize the molecular mechanisms involved and to better understand the whole set of sequelae of this zoonotic disease. A potential limitation of the study is related to the mixed species composition from which vesicles were isolated. However, molecular identification cannot be performed prior to incubation, and the contribution of different species other than the most prevalent (*A. pegreffii*) is likely to be negligible. Moreover, mixed infections in humans cannot be ruled out, thus making our setting representative of real human infections, especially in areas where the two species coexist. As for miRNA's biological role in modulating the transcripts availability of the downregulated genes, approaches such as transfection or antisense oligos appear needed to confirm their effect.

Another potential limitation of the study relies on the administration of an enriched EV fraction isolated with a precipitation-based kit, which also concentrates particles of the same size range together with EVs, and even free proteins. In future studies, additional isolation methods, such as size-exclusion chromatography, will be implemented to confirm the results obtained here.

## Supplementary Information

Additional file 1: Additional material 1: Experimental and analytical details about iNTA. Figure S1: Finite track length adjustmentimage for nanoparticle tracking analysisof extracellular vesicles secreted by third-stage larvae of *Anisakis* spp.. The concentration and size were obtained in the two experimental settings with pools of 20and 50L3. Figure S2: iNTA scatter plot of the two experimental settings of *Anisakis* EVs.andPools of 50 L3;andpools of 20 L3. Color bars denote point density. The red lines correspond to the scenario of a shell thickness of 10 nm and shell refractive index of 1.36. The inner protein non-water content varies from the bottom to top line as 0%, 10%, 25%, 50%, 75%, 100%. Figure S3: Distribution of diameter values from the three approaches used to characterize EVs, with size in nm along the x-axis and number of measurements in the y-axis. Figure S4: RNA-seq sample correlation analyses to test the effect of covariation and batch effect, based on PCA of the distance matrixand FPKM. Figure S5: *Anisakis* extracellular vesiclemodulation of cytokine gene expression in 2D cultures of human intestinal organoids after 48 h of exposure.Il8 gene expression in HIO;Il33 gene expression in HIO;Il1β gene expression in HIO. Data are expressed as a fold change compared to the control samples and as means ± SEM. Table S1: Mean concentration and median size including interquartile rangeof the two different classes of nanoparticles measured using NTA and iNTA. Refractive index is also shown for iNTA. For concentration, the standard deviation of two successive measurements is indicated. Table S2: Primer list used for qRT-PCR with indication of target, nucleotide sequence of forward and reverse primers, bibliographic references and efficiency value obtained for standard curves.

Additional file 2: List of top100 most abundant trascripts in the samples, with information on transcript IDand the relative description, according to Genecard and Uniprot databases. Panther gene list—molecular function details on the three most represented hits among the top100 most abundant transcripts in the samples

Additional file 3: List of differentially expressed geneswith associated statistics; list of non-significant upregulated transcripts in *Anisakis* EV-treated human intestinal organoidswith Panther analyses; list of non-significant downregulated transcripts in *Anisakis* EV-treated human intestinal organoidswith Panther analyses.

## Data Availability

Details on EVs are available at the EVtrack website under the ID EV240048. Raw RNAseq data are available at the SRA website under the Bioproject PRJNA942614. Any query for material and correspondence should be sent to SC (serena.cavallero@uniroma1.it).

## Abbreviations

CEACAM: Carcinoembryonic antigen-related cell adhesion molecule
CXCL: Chemokine (C-X-C motif) ligand
DEELISA: Enzyme-linked immunosorbent assay
EPHB2: Ephrin type-B receptor 2 gene
EVs: Extracellular vesicles
FC: Fold-change
FDR: False discovery rate
GM-CSF: Granulocyte-macrophage colony-stimulating factor
HIO: Human intestinal organoids
HOC: Human organoids controls
HOT: Human organoids treated with *Anisakis* EVs
IL: Interleukin encoding genes
iNTA: Interferometric nanoparticle tracking analysis
L3: Third-stage larva
LEFTY: Left–right determination factors 1
miRNAs: Micro-RNAs
NTA: Nanoparticle tracking analysis
NUPR1: Nuclear protein 1 transcription regulator gene
qRT-PCR: Quantitative real-time polymerase chain reaction
RI: Refractive index
SEM: Scanning electron microscopy
TACC1: Transforming acidic coiled-coil containing protein 1 gene
TEM: Transmission electron microscopy
