# Supplementary material for: Anisakis extracellular vesicles elicit immunomodulatory and potentially tumorigenic outcomes on human intestinal organoids
Source: Parasit Vectors. 2024 Sep 17;17:393. doi: 10.1186/s13071-024-06471-7 (PMC11406850; doi:10.1186/s13071-024-06471-7)
Supplement: Supplementary file 1 — Additional file 1: Additional material 1: Experimental and analytical details about iNTA. Figure S1: Finite track length adjustmentimage for nanoparticle tracking analysisof extracellular vesicles secreted by third-stage larvae of Anisakis spp.. The concentration and size were obtained in the two experimental settings with pools of 20and 50L3. Figure S2: iNTA scatter plot of the two experimental settings of Anisakis EVs.andPools of 50 L3;andpools of 20 L3. Color bars denote point density. The red lines correspond to the scenario of a shell thickness of 10 nm and shell refractive index of 1.36. The inner protein non-water content varies from the bottom to top line as 0%, 10%, 25%, 50%, 75%, 100%. Figure S3: Distribution of diameter values from the three approaches used to characterize EVs, with size in nm along the x-axis and number of measurements in the y-axis. Figure S4: RNA-seq sample correlation analyses to test the effect of covariation and batch effect, based on PCA of the distance matrixand FPKM. Figure S5: Anisakis extracellular vesiclemodulation of cytokine gene expression in 2D cultures of human intestinal organoids after 48 h of exposure.Il8 gene expression in HIO;Il33 gene expression in HIO;Il1β gene expression in HIO. Data are expressed as a fold change compared to the control samples and as means ± SEM. Table S1: Mean concentration and median size including interquartile rangeof the two different classes of nanoparticles measured using NTA and iNTA. Refractive index is also shown for iNTA. For concentration, the standard deviation of two successive measurements is indicated. Table S2: Primer list used for qRT-PCR with indication of target, nucleotide sequence of forward and reverse primers, bibliographic references and efficiency value obtained for standard curves. [file 13071_2024_6471_MOESM1_ESM.docx]

**Supplementary material.**

**Additional material 1. Experimental and analytical details about iNTA.** reads were performed using plasma cleaned (1 minute in oxygen plasma at 500 W) chambered cover glasses (IBIDI µ-Slide 18 well with glass bottom), then 60 µm of 40 nm GNPs from BBI Solutions diluted 1:200 in milliQ water were introduced into one of the wells, used as a reference to set the focus correctly. The chambered cover glasses were passivated (the coverglasses were plasma cleaned 1 minute in oxygen plasma at 500 W). Wells were filled with 100 µL of 10 mg/mL mPEG2000-Silane dissolved in PEG solution (95% Ethanol (v/v), 5% milliQ, pH was set to 2.0 with 1 M HCl). The chambered coverglass was incubated at 50° C for solution evaporation, sonicated for 10 minutes in milliQ water and blow dried with nitrogen gas. Passivated cover glasses were used the same day. The measurement setup was similar to a previously described (Kashkanova et al 2022). Here, a Leica HC PL APO 160x/1.43 Oil microscope objective was used for a pixel size of 70.6 nm and a field of illumination (FOV) of 5.2 x 5.2 µm^2^. A uniform illumination was achieved by employing Acousto-Optical Deflectors (AOD) in the incident beam path, scanning at a rate ~10x faster than the acquisition rate. We measure at 10 kHz with 50 µs exposure time. The measurement and analysis procedure is the same as described previously (Kashkanova et al 2022). The microscope focus was set at 1 µm above the coverglass. Videos of particles diffusing in 50 µL volumes of fluid inside individual IBIDI wells were recorded for 5 minutes 2-8 times using pylablib cam-control (doi: 10.5281/zenodo.7324876). The illumination intensity was constant for all the measurements. The videos were analyzed by applying median background correction and radial variance transform, and contrast to back-scattering cross-section calibration were performed as described before (Kashkanova et al 2022). Particles were tracked using the trackpy python package (Allan et al 2021) with a linking radius of 8 px = 565 nm. The particle was allowed to disappear for at most 20 frames before the trajectory would get a new identifier. Only the particles for which the central maximum could be fit with a Gaussian and the width of the central maximum at the position of the maximum contrast was between 100 and 120 nm were considered for further analyses, as for those the scattering cross-section could be extracted accurately. This time we used polystyrene beads and required mean RI to be 1.6. This results in calibration factor β=4.5x10^7^ m^-1^.

**Supplementary Figures.**

**Figure S1:** Finite track length adjustment (FTLA) image for nanoparticle tracking analysis (NTA) of extracellular vesicles secreted by third-stage larvae of *Anisakis* spp. (*Anisakis simplex* sensu stricto, *Anisakis pegreffii*, and the hybrid form). The concentration and size were obtained in the two experimental settings with pools of 20 a) and 50 b) L3 (the number of particles is intended as ˆ10).

a)
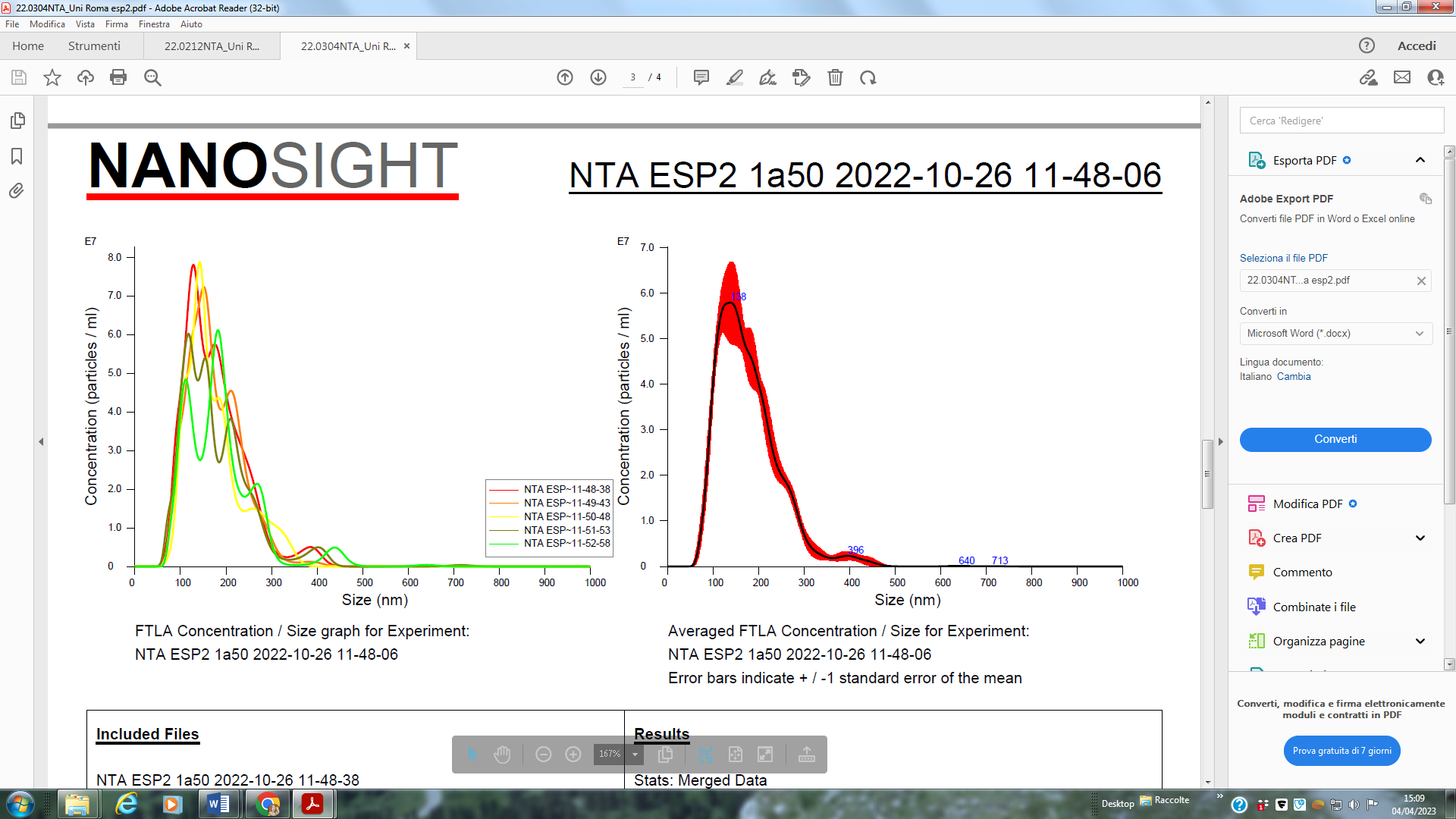
b)
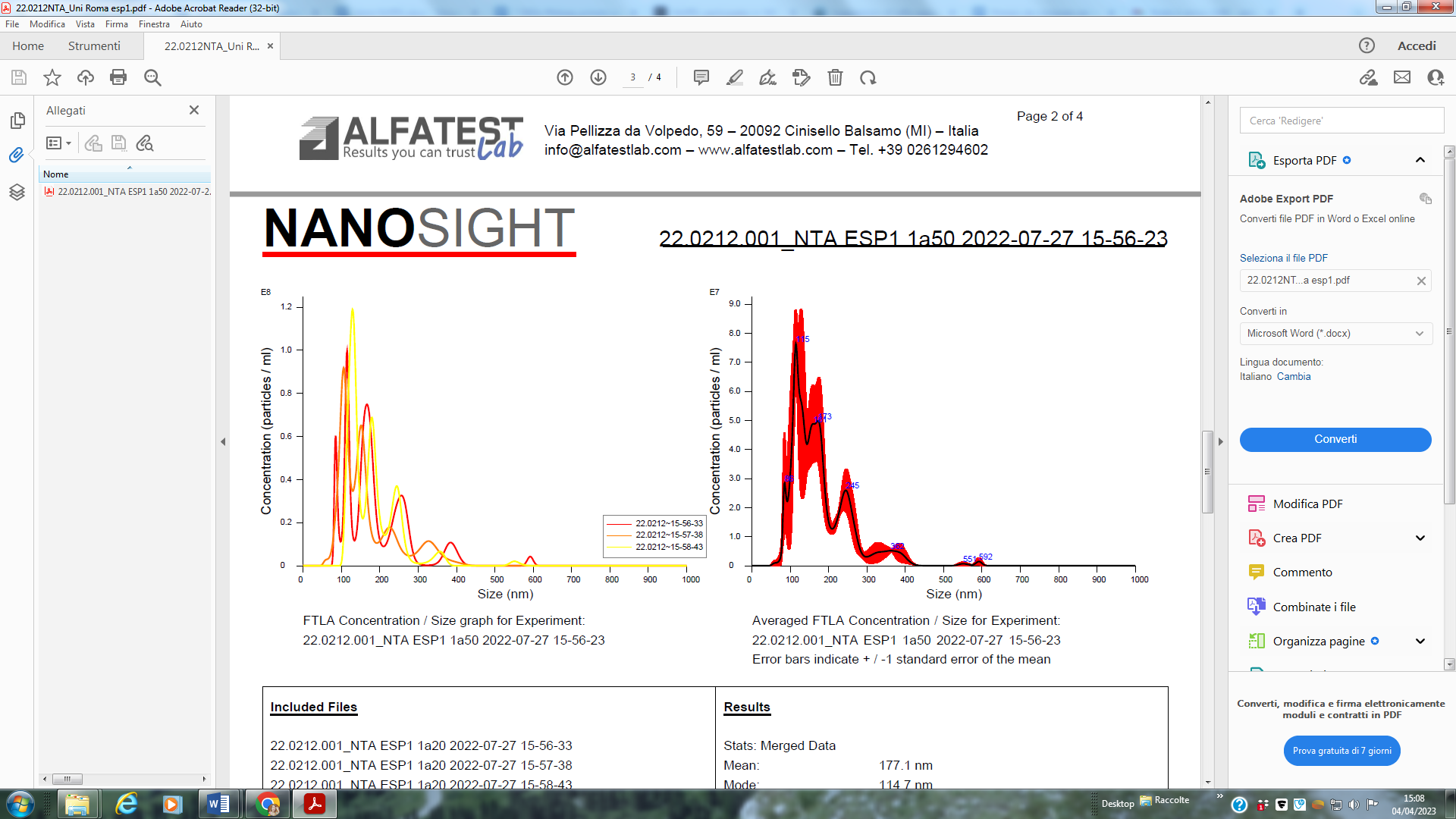


**Figure S2:** iNTA scatter plot of the two experimental settings of *Anisakis* EVs, a) and c): pools of 50 L3; b) and d): pools of 20 L3. Color bars denote point density. The red lines correspond to the scenario of a shell thickness of 10 nm and a shell refractive index of 1.36. The inner protein non-water content varies from bottom line to the top line as 0%, 10%, 25%,50%, 75%, 100%.

a)
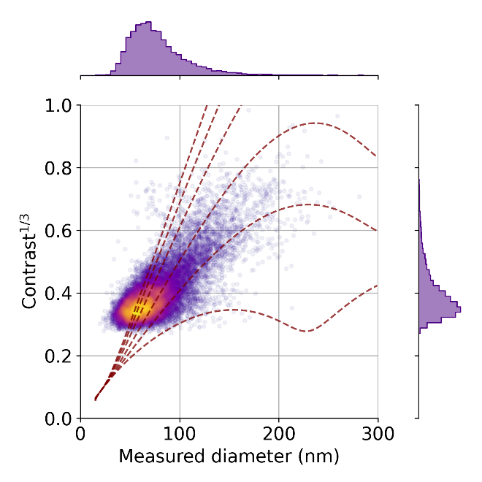
 b)
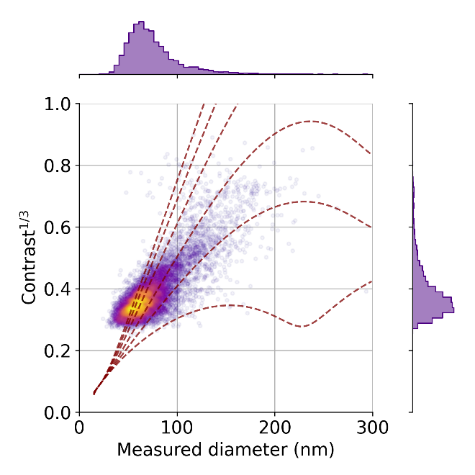
.

c)
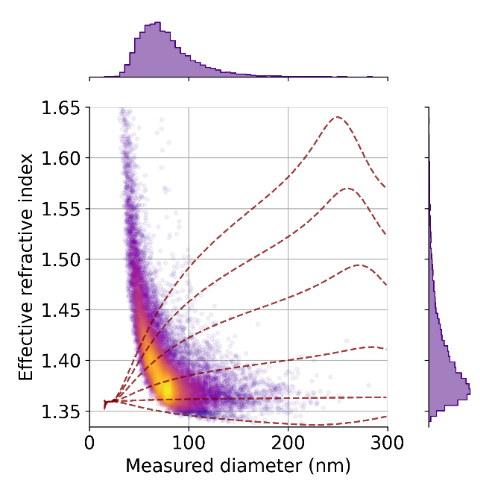
d)
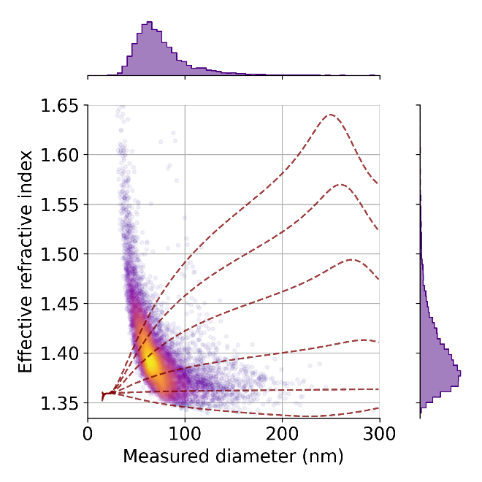


**Figure S3:** Distribution of diameters values from the three approaches used to characterize EVs (TEM, iNTA and NTA), with size in nm along the x-axis and number of measurements in the y-axis.


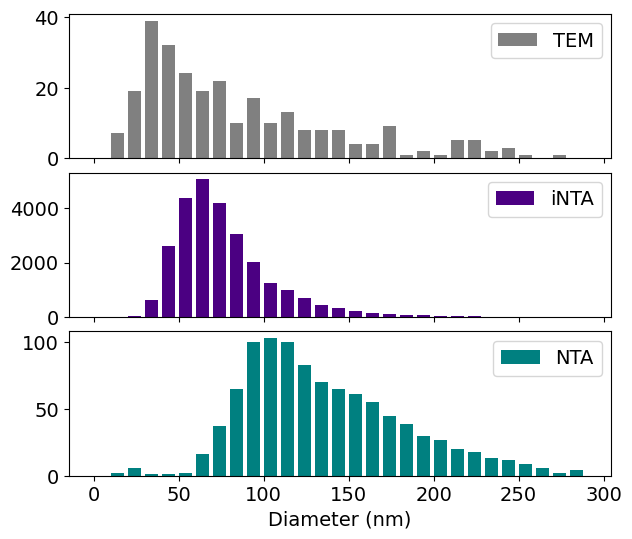


**Figure S4:** RNA-seq sample correlation analyses to test the effect of covariation and batch effect, based on PCA of the distance matrix a) and of the FPKM b).

a)
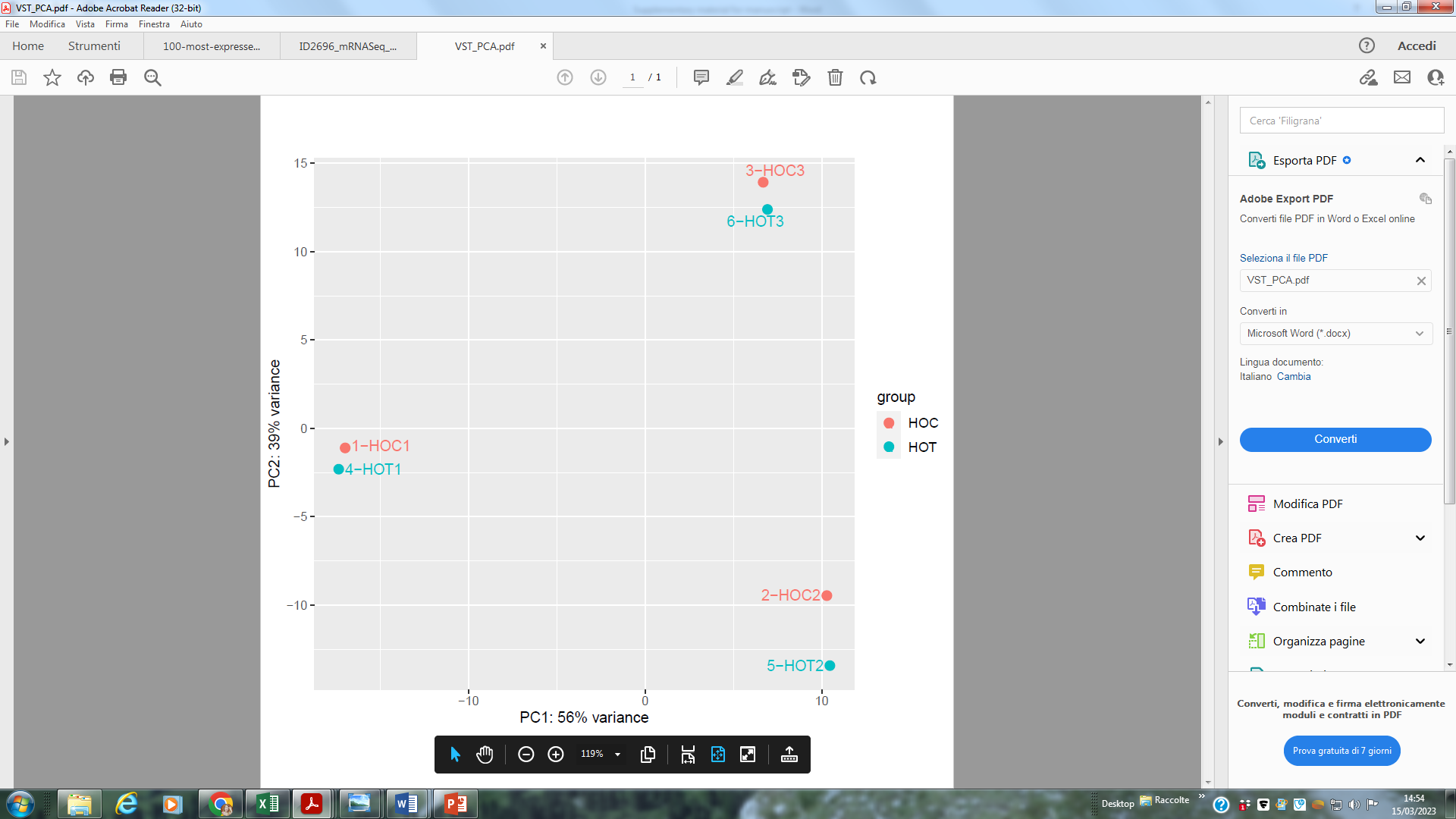
b)
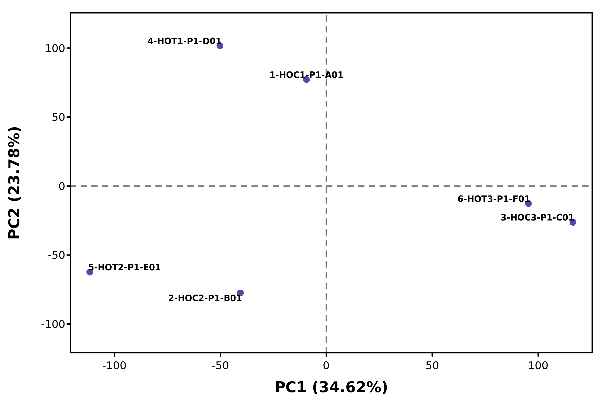


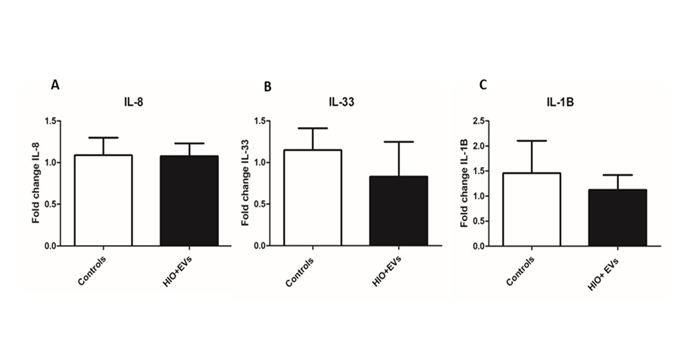


**Figure S5: *Anisakis* extracellular vesicles (EVs) modulation of cytokines gene expression in 2D-cultures of human intestinal organoids after 48h of exposure.** A) Il8 gene expression in HIO; B) Il33 gene expression in HIO and C) Il1β gene expression in HIO. Data are expressed as a fold change compared to the control samples and as means ± SEM (standard error mean).

**Supplementary Table.**

**Table S1**: Mean concentration and median size including interquartile range (IQR) of the two different classes of nanoparticles measured using NTA and iNTA. Refractive index is also shown for iNTA. For concentration, the standard deviation of two successive measurements is indicated.

|  | **NTA** | | **iNTA** | | |
| --- | --- | --- | --- | --- | --- |
| **Sample** | Mean  concentration  [particles/mL] | Median  size (IQR)  [nm] | Mean  concentration  [particles/mL] | Median  size (IQR)  [nm] | Median  RI (IQR) |
| **Ev from 50 L3** | 7,83x10^9^ | 161,5  (104,3-267,4) | 4,6x10^11^ | 72  (58-91) | 1,39  (1,38-1,42) |
| **Ev from 20 L3** | 2,49x10^10^ | 129,3  (81,7-218,1) | 1,4x10^11^ | 67  (56-83) | 1,39  (1,37-1,42) |

**Table S2**: **Summary of the RNA-seq data obtained for human intestinal organoids.** Sample ID (controls=HOC and *Anisakis* EV-treated=HOT) with the number of obtained reads, the reads passed the quality check and the percentage of reads mapping to the hg38-iGenomes version and those uniquely aligned (expressed in million).

| **Sample_ID** | **Obtained**  **reads** | **Filtered**  **reads** | **% map to**  **hg38-iGenomes** | **Uniquely**  **aligned** |
| --- | --- | --- | --- | --- |
| HOC1 | 97.17 | 96.9 | 89,9% | 43.5 |
| HOC2 | 68.63 | 68.2 | 88,5% | 30.2 |
| HOC3 | 96.30 | 95.7 | 88,6% | 42.4 |
| HOT1 | 55.88 | 55.5 | 86,3% | 24.0 |
| HOT2 | 56.82 | 56.6 | 86,9% | 24.6 |
| HOT3 | 67.86 | 67.5 | 86,2% | 29.1 |

**Table S3**:primers list used for qRT-PCR with indication of target, nucleotide sequence of forward and reverse primers, bibliographic references and efficiency value obtained for standard curves.

| **Target** | **Primer F** | **Primer R** | **References** | **Efficiency in**  **Standard curves** |
| --- | --- | --- | --- | --- |
| GAPDH | TGTTGCCATCAATGACCCCTT | CTCCACGACGTACTCAGCG | Borkowski et al 2014 | 100% |
| Il1b | CTCTCTCACCTCTCCTACTCAC | ACACTGCTACTTCTTGCCCC | Tiwari et al 2011 | 112% |
| Il8 | CTCTTGGCAGCCTTCCTGATTT | TGGGGTGGAAAGGTTTGGAGTA | Borkowski et al 2014 | 111% |
| Il33 | AATCAGGTGACGGTGTTG | ACACTCCAGGCTCAGTCTTG | Perez et al 2020 | 102% |
| NUPR1 | CTGGATGAATCTGACCTCTA | CGCTTCTTCCTCTCTGAATT | Jiang et al., 2021 | 111% |
| EPHB2 | GCAGTGTCCATCATGCATC | AGTACTGCAGCTCATAGTCC | Sakamoto et al., 2012 | 100% |
| LEFTY | AGGTTCAGCCAGAGCTTCC | CACCAGCAGGTGTGTGCT | Saito et al 2013 | 98% |
| TACC1 | AACTCCCCACCCCTCTCTT | CTTCTTCACCTTACAGCCACTC | Partheen et al., 2006 | 102% |

Borkowski J, Li L, Steinmann U, Quednau N, Stump-Guthier C, Weiss C, Findeisen P, Gretz N, Ishikawa H, Tenenbaum T, Schroten H, Schwerk C. *Neisseria meningitidis* elicits a pro-inflammatory response involving IκBζ in a human blood-cerebrospinal fluid barrier model. *J Neuroinflammation*. 2014; 13, 11:163.

Jiang L, Wang W, Li Z, Zhao Y, Qin Z. NUPR1 participates in YAP-mediate gastric cancer malignancy and drug resistance via AKT and p21 activation*. J Pharm Pharmacol.* 2021; 73, 6:740.

Partheen K, Levan K, Österberg L, Horvath G. Expression analysis of stage III serous ovarian adenocarcinoma distinguishes a sub-group of survivors. *European J Cancer*, 2006; 42, 16:2846.

Perez F, Ruera CN, Miculan E, Carasi P, Dubois-Camacho K, Garbi L, Guzman L, Hermoso MA and Chirdo FG. IL-33 Alarmin and Its Active Proinflammatory Fragments Are Released in Small Intestine in Celiac Disease. *Front. Immunol*. 2020 11:581445.

Tiwari RL, Singh V, Singh A, Kumar Barthwal M. IL-1R–Associated Kinase-1 Mediates Protein Kinase Cδ-Induced IL-1β Production in Monocytes. *J Immunol.* 2011; 187, 5:2632.

Saito, A, Ochiai, H, Okada, S, Miyata, N, Azuma T. Suppression of Lefty expression in induced pluripotent cancer cells. *The FASEB Journal*, 2013; 27: 2165.
